# Pneumococcal Conjugate Vaccines and Otitis Media

**DOI:** 10.1155/2012/764573

**Published:** 2012-11-13

**Authors:** Sylvia Taylor, Paola Marchisio, Anne Vergison, William P. Hausdorff, Mark Haggard

**Affiliations:** ^1^Global Vaccine Development, GlaxoSmithKline Vaccines, 1300 Wavre, Belgium; ^2^Department of Pathophysiology and Transplantation, University of Milan and Fondazione IRCCS Cà, Granda Ospedale Maggiore Policlinico, 20122 Milan, Italy; ^3^Paediatric Infectious Diseases, Infection Control and Epidemiology Unit, Université Libre de Bruxelles, 1020 Brussels, Belgium; ^4^Multi-Centre Otitis Media Study Group, Department of Psychology, University of Cambridge, Cambridge CB2 3EB, UK

In a recent review published in International Journal of Otolaryngology, Fletcher and Fritzell provide a comprehensive and useful summary of the clinical trials assessing efficacy of pneumococcal conjugate vaccines (PCVs) against otitis media (OM) [1]. Although the Results section of the review is limited to clinical trial data, the Discussion section also includes interpretation of PCV “real-life impact” data derived from post-implementation database studies. We have some reservations about how these data are often interpreted that may affect Fletcher and Fritzell's overall conclusions. These points are stated more fully in our systematic review of PCV efficacy and effectiveness data published [2] around the same time as those authors' contribution.

Fletcher and Fritzell provide a large study, but limited to private insurance data, in children aged <2 years in the United States (USA) as their prime example of PCV effectiveness [3]. However, this study was, at the time of Fletcher and Fritzell's publication, only one of seven US studies assessing the impact of universal mass vaccination with a 7-valent PCV (7vCRM) on OM visit rates [3–9]. Results of the Zhou et al. study are distinct from the others in two main ways.

1. Zhou et al. observed a 43% decrease in OM visit rates, whereas the other studies reported a range from 37% decrease to 7% increase. The two large population-based studies analyzing nationally representative samples showed a strong central tendency: 12% decrease in <2 year olds [5] and 13% decrease in <5 year olds [4].
2. Of the seven studies, only Zhou et al. observed that OM visit rates were increasing (+14%) over the three to five years before 7vCRM introduction rather than already decreasing (by 16–24%).

It is thus of concern that only an outlier study in its category received such exclusive attention. 

There also appears to be a slight error (page 12, paragraph 1) in the calculation of number of annual OM visits prevented per 100 children aged <2 years based on the Zhou et al. data (page 255, paragraph 5, and Figure 1), which indicate that, compared to 1997–1999, the annual OM visit rate in 2004 had decreased by 929 visits per 1000 person-years and that this decrease in annual rate had already been essentially achieved by 2002. These many visits prevented *every* year from 2002 to 2004 would mean a 7-8-fold higher effectiveness estimate than the 23 annual visits per 100 children calculated by Fletcher and Fritzell or based on the cited vaccine coverage rates of 41% in 2002, 68% in 2003, and 73% in 2004, an implausible 163 OM visits prevented per 100 children vaccinated.

Given the already decreasing rate of OM visits generally observed before 7vCRM introduction in USA and elsewhere, it is important to consider pre-/post-introduction comparisons by assessing confounding trends over time that may have biased the estimates, particularly if these are more favourable to the vaccine. A Canadian study [10] adjusted for trend over time using time-series regression, which altered the raw decrease in OM visit rates from 25% down to 13% attributable to 7vCRM when adjusted. A rise over time in family thresholds for consultation or in physician thresholds for diagnosis and reporting OM in an era of increasing use of guidelines [11] and a role for watchful waiting cannot be ruled out as a main basis of the trends over time seen. However, there are also other credible risk factors for OM known to have undergone secular changes through the period considered such as a shift of practice to higher antibiotic dosage (or the doubling of long-acting macrolide use) in US children around the same time as 7vCRM introduction or decreased exposure of children to passive smoking [2].

The authors also briefly cite one non-US impact study [12], which reported a 38% decrease in otorrhea rates per emergency department visit in Greece. However, this drop is not credibly attributable to vaccination because it occurred 1-2 years before mass pneumococcal vaccination with 7vCRM in Greece and no further drop was observed when mass vaccination was eventually introduced in Greece in 2006. A large drop could not have been due to the prior, presumably minimal, private market use. Furthermore, the proportion of pneumococcal otorrhea episodes due to vaccine serotypes remained high in the period following the decrease whereas a 1–3-year decline phase for these serotypes (from growing herd protection) would have been required for vaccination to constitute a valid explanation of the data. The initial decline is instead likely to be primarily accounted for by nonvaccine factors such as sampling artefact.

We thus urge those interested in PCV real-life impact on OM to fully consider the methodological limitations of the studies published so far and use a more analytic and fully representative approach to synthesizing the evidence. We agree with Fletcher and Fritzell that 7vCRM is efficacious and moderately effective in preventing OM, but we consider it essential to avoid overstating the magnitude of effect.
